# Environmental Characteristics and Anthropogenic Impact Jointly Modify Aquatic Macrophyte Species Diversity

**DOI:** 10.3389/fpls.2018.01001

**Published:** 2018-08-10

**Authors:** Merja Elo, Janne Alahuhta, Antti Kanninen, Kristian K. Meissner, Katri Seppälä, Mikko Mönkkönen

**Affiliations:** ^1^Department of Biological and Environmental Science, University of Jyväskylä, Jyväskylä, Finland; ^2^School of Resource Wisdom, University of Jyväskylä, Jyväskylä, Finland; ^3^Geography Research Unit, University of Oulu, Oulu, Finland; ^4^River Basin Management Unit, Freshwater Centre, Finnish Environment Institute, Helsinki, Finland; ^5^Centre for Economic Development, Transport and the Environment for North Savo, Turku, Finland; ^6^Programme for Environmental Information, Finnish Environment Institute SYKE, Jyväskylä, Finland; ^7^Eawag, Swiss Federal Institute of Aquatic Science and Technology, Dübendorf, Switzerland

**Keywords:** biodiversity, beta diversity, community composition, eutrophication, human impact, null models species richness, water plants

## Abstract

Species richness and spatial variation in community composition (i.e., beta diversity) are key measures of biodiversity. They are largely determined by natural factors, but also increasingly affected by anthropogenic factors. Thus, there is a need for a clear understanding of the human impact on species richness and beta diversity, the underlying mechanisms, and whether human-induced changes can override natural patterns. Here, we dissect the patterns of species richness, community composition and beta diversity in relation to different environmental factors as well as human impact in one framework: aquatic macrophytes in 66 boreal lakes in Eastern Finland. The lakes had been classified as having high, good or moderate status (according to ecological classification of surface waters in Finland) reflecting multifaceted human impact. We used generalized least square models to study the association between different environmental variables (Secchi depth, irregularity of the shoreline, total phosphorus, pH, alkalinity, conductivity) and species richness. We tested the null hypothesis that the observed community composition can be explained by random distribution of species. We used multivariate distance matrix regression to test the effect of each environmental variable on community composition, and distance-based test for homogeneity of multivariate dispersion to test whether lakes classified as high, good or moderate status have different beta diversity. We showed that environmental drivers of species richness and community composition were largely similar, although dependent on the particular life-form group studied. The most important ones were characteristics of water quality (pH, alkalinity, conductivity) and irregularity of the shoreline. Differences in community composition were related to environmental variables independently of species richness. Species richness was higher in lakes with higher levels of human impact. Lakes with different levels of human impact had different community composition. Between-lake beta diversity did not differ in high, good or moderate status groups. However, the variation in environmental variables shaping community composition was larger in lakes with moderate status compared to other lakes. Hence, beta diversity in lakes with moderate status was smaller than what could be expected on the basis of these environmental characteristics. This could be interpreted as homogenization.

## Introduction

Anthropogenic impacts have reached a point where every corner of our planet is somehow affected by human actions (Millennium Ecosystem Assesment, 2005). Our understanding of how these actions affect biodiversity is hampered by, for instance, the measurement of different aspects of biodiversity (McGill et al., 2015). The most intriguing measure of biodiversity is species richness. It has been commonly used as an ecological indicator as well as a decision variable in setting conservation targets (Myers et al., 2000). Consequently, a myriad of studies have been performed in order to reveal the anthropogenic impact on species richness across organisms and ecosystems (Murphy and Romanuk, 2014). Perhaps surprisingly, two large meta-analyses support the conclusion that while changes in local species richness due to human actions range from positive to negative, in general human actions does not lead in decreased species richness (Vellend et al., 2013; Dornelas et al., 2014; but see Gonzalez et al., 2016).

No change in species richness may be accompanied by significant temporal (or spatial) changes in community composition, generally referred as beta diversity (Sax and Gaines, 2003; Dornelas et al., 2014). Thus, although the number of species in a community does not change, the identities of species forming the community may alter. Increase in beta diversity refers to less similar species composition among communities whereas decrease in beta diversity refers to more uniform species compositions among communities. Decrease in beta diversity may potentially lead to homogenization of biotas where already abundant species colonize new environments and often replace rare species (McKinney and Lockwood, 1999).

Species richness shows three well-known natural patterns: the number of species tend to increase with area (Connor and McCoy, 1979; Lomolino, 2000), habitat heterogeneity (Hurlbert, 2004) and factors related to energy (Wright, 1983; Field et al., 2009). Similarly, beta diversity has been shown to vary with area (Heegaard, 2004), habitat heterogeneity (Jones et al., 2003) and energy (Qian and Xiao, 2012). Thus, these two components of biodiversity reflect congruent macroecological patterns, although this congruency can also partly be explained by the fact that many measures of beta diversity are not independent of species richness (e.g., Legendre, 2014).

How and to what extent human actions affect species richness and beta diversity depend on the processes underlying community assembly and how human impact intervene with them. Community assembly processes has been divided to (a) deterministic, niche-based processes, such as environmental filtering and biotic interactions, and to (b) neutral, stochastic processes, such as chance colonization, random extinctions and ecological drift (Chase and Myers, 2011). Although the relative importance of these processes on community assemblages is a subject of debate, there is a growing consensus that both are important (Vellend, 2010; Chase and Myers, 2011), and that the study of beta diversity may result in important insights into these processes (Chase and Myers, 2011). In summary, studying the mechanisms behind human induced changes in species richness and beta diversity is needed.

Here, we try to resolve the complex picture by dissecting the patterns of species richness, community composition and beta diversity in relation to different environmental factors as well as human impact in one framework: aquatic macrophytes in 66 boreal lakes. The lakes are classified having either a high, good or moderate status *sensu* the EU Water Framework Directive (Directive 2000/60/EC; see Methods). The major human impact on aquatic macrophyte communities in freshwaters, and particularly to our naturally oligotrophic study lakes, is eutrophication due to leakage of nutrients from agricultural land. Species richness of aquatic macrophytes has been found to have unimodal response to primary productivity (Dodson et al., 2000) but in Finnish naturally oligotrophic lakes eutrophication typically increases species richness of aquatic flora (Leka et al., 2008). Besides nutrient levels, species richness of aquatic macrophytes has also been previously related to other chemical properties of water, lake area and habitat heterogeneity (Toivonen and Huttunen, 1995; Vestergaard and Sand-Jensen, 2000; Alahuhta et al., 2013; Alahuhta, 2015). In addition, community composition as well as beta diversity of aquatic macrophytes has been shown to be shaped by local environmental factors such as habitat heterogeneity and water quality whilst dispersal seems to play a minor role, at least at regional extents considered here (Alahuhta et al., 2013; Viana et al., 2016).

We specifically study (i) the main environmental drivers of species richness and community composition, (ii) whether these drivers are shared for both metrics, (iii) if differences in community composition are related to environmental variables, independently of species richness, indicating deterministic processes or (iv) if these differences are simply attributed to neutral processes. Finally, we address (v) whether beta diversity varies among the lakes under different levels of human impact. Beta diversity, defined as spatial or temporal change in community composition, can refer to both directional turnover along an environmental gradient or non-directional variation, i.e., variation in community compositions among sites within a group (Anderson et al., 2011). Thus, change in the *mean* community composition could be interpreted as beta diversity but to avoid confusion we use the term “beta diversity” to refer to non-directional variation in community composition among lakes having similar level of human impact (i.e., *dispersion* in community composition; Anderson et al., 2006).

Following Alahuhta et al. (2013), we predict that the same environmental drivers are responsible for both species richness and community composition, and that differences in community composition are due to deterministic processes rather than neutral processes. The changes on aquatic macrophyte communities depend on the original state of the lake as well as the level of human impact. For the naturally oligotrophic lakes where the level of human impact ranging from low to moderate, we predict that species richness is highest in lakes with relatively high human impact (moderate status), lower in lakes with lower human impact (good status) and lowest in lakes with relatively low human impact (high status). Finally, we predict that lakes show distinct communities according to the level of human impact and that beta diversity among lakes with moderate human impact is smaller compared to that in lakes with lower levels of human impact (Lougheed et al., 2008).

The novelty in our study is that we use null model approach to investigate the effects of environmental variation and human impact using comparable methods across different response variables in the same research setting. These response variables consist of different measures of biodiversity (species richness, community composition, beta diversity) and multiple life-form groups. Thus, although some of the results are similar to what earlier papers have found (e.g., Declerck et al., 2005; Alahuhta et al., 2013; Wezel et al., 2014), we seek to provide a more comprehensive and general picture of multiple drivers and their relative strengths of macrophyte diversity than previously evidenced.

## Materials and methods

### Field work and study variables

**Figure 1:**
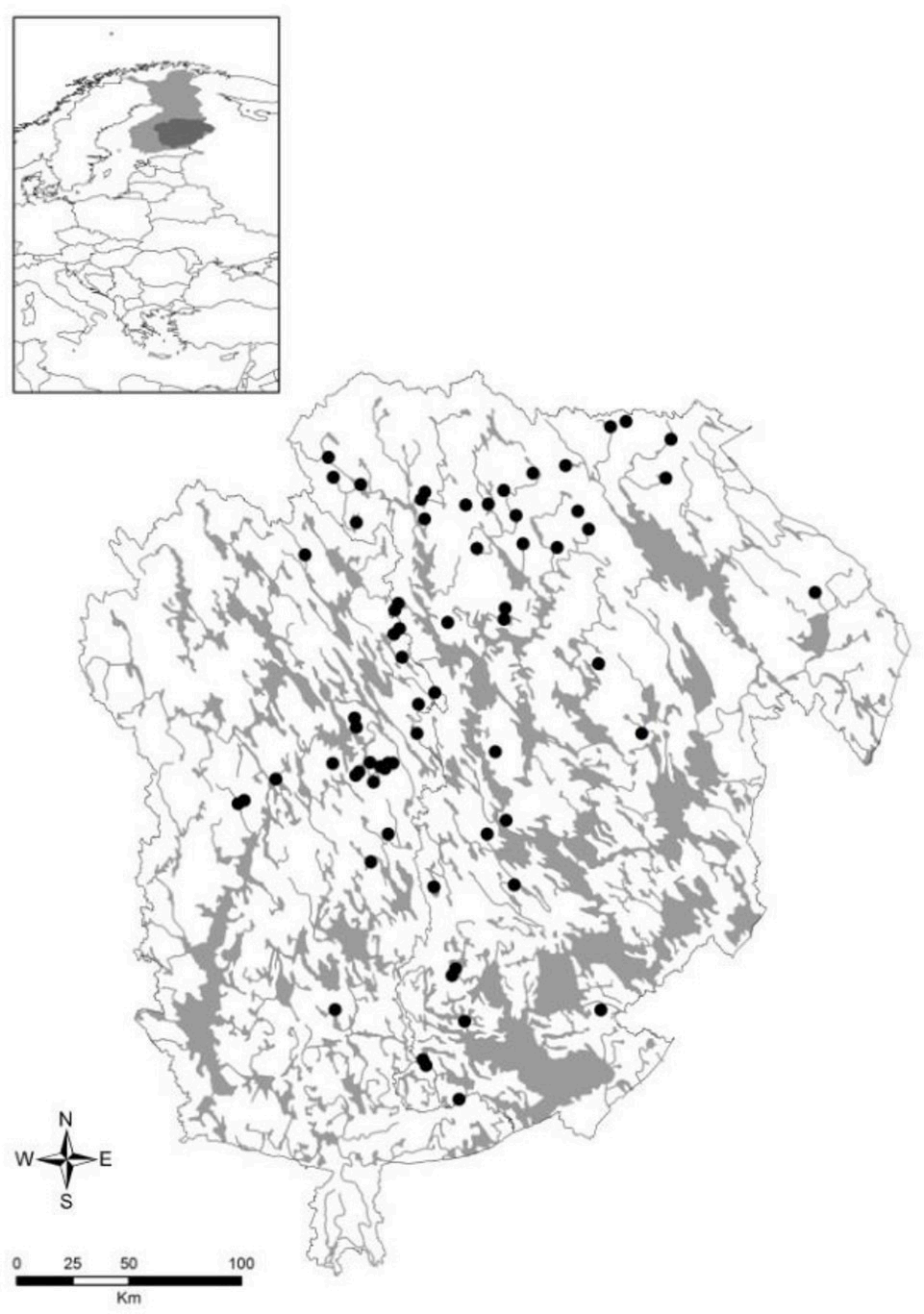
Study lakes (*n* = 66) are situated in Eastern Finland representing geographical variation from 61°9′ N to 63°49′ N and 25°44′ W to 30°50′ W.

The 66 study lakes are situated in Eastern Finland (Figure 1). The aquatic macrophyte species data was collected by regional environmental authorities (e.g., Kanninen et al., 2009). The field surveys were conducted using the main belt transect method (Kanninen et al., 2013) between June and September of 2002–2008. The 5-meter wide transect started from the upper eulittoral and continued to the outer borderline of submersed vegetation, or to the middle point of the basin if vegetation extended over the entire lake. Transect was divided into zones according to the dominant life-form or species. Macrophytes were observed by wading or by boat, with the aid of rake and hydroscope. The species were listed and visual abundance estimates (frequency and coverage) were made for each taxon. The number of transects per lake varied (mean = 15.5, *SD* = 4.98, range = 7–43) according to lake size and morphology.

A total of 104 aquatic macrophyte species were recorded. Based on their structural and functional similarities we combined individual growth forms (Toivonen and Huttunen, 1995) to 4 life-form groups (shore plants, helophytes, rhizophytes, pleustophytes). We explored the effect of different explanatory variables on 5 different species richness estimates: (1) total macrophyte species richness (104 species), (2) species richness of shore plants (26 species), (3) species richness of helophytes (plants that are rooted in sediments or soils that are periodically inundated, with foliage extending into the air; 19 species), (4) species richness of rhizophytes (hydrophyte species attached to substrate): floating-leaved species, isoetids, elodeids, charophytes (49 species), and (5) species richness of pleustophytes (free-floating species): lemnids and ceratophyllids (10 species). All study species and their grouping are presented in Table 1.

**Table 1:** Macrophyte species, their growth form (*sensu* Toivonen and Huttunen 1995), and life-form group used in the current study.

**Species**	**Growth form**	**Life-form group**
*Hydrocharis morsus-ranae* L.	Lemnid	Free-floating species
*Lemna minor* L.	Lemnid	Free-floating species
*Spirodela polyrhiza* (L.) Schleid.	Lemnid	Free-floating species
*Ceratophyllum demersum* L.	Ceratophyllid	Free-floating species
*Stratiotes aloides* L.	Ceratophyllid	Free-floating species
*Utricularia australis* R. Br.	Ceratophyllid	Free-floating species
*Utricularia intermedia* Hayne	Ceratophyllid	Free-floating species
*Utricularia minor* L.	Ceratophyllid	Free-floating species
*Utricularia ochroleuca* R. W. Hartm	Ceratophyllid	Free-floating species
*Utricularia vulgaris* L.	Ceratophyllid	Free-floating species
*Callitriche hermaphroditica* L.	Elodeid	Rhizophyte
*Elodea canadensis* Michx.	Elodeid	Rhizophyte
*Myriophyllum alterniflorum* DC.	Elodeid	Rhizophyte
*Myriophyllum sibiricum* Kom.	Elodeid	Rhizophyte
*Myriophyllum verticillatum* L.	Elodeid	Rhizophyte
*Najas flexilis* (Willd.) Rostk. & W. L. E. Schmidt	Elodeid	Rhizophyte
*Potamogeton alpinus* Balb.	Elodeid	Rhizophyte
*Potamogeton berchtoldii* Fieber	Elodeid	Rhizophyte
*Potamogeton compressus* L.	Elodeid	Rhizophyte
*Potamogeton gramineus* L.	Elodeid	Rhizophyte
*Potamogeton gramineus x perfoliatus*	Elodeid	Rhizophyte
*Potamogeton obtusifolius* Mert. & W. D. J. Koch	Elodeid	Rhizophyte
*Potamogeton perfoliatus* L.	Elodeid	Rhizophyte
*Potamogeton praelongus* Wulfen	Elodeid	Rhizophyte
*Ranunculus peltatus* Schrank	Elodeid	Rhizophyte
*Ranunculus peltatus ssp. baudotii* (Gordon) C. D. K. Cook	Elodeid	Rhizophyte
*Ranunculus peltatus ssp. peltatus*	Elodeid	Rhizophyte
*Callitriche cophocarpa* Sendtn.	Elodeid	Rhizophyte
*Callitriche palustris* L.	Elodeid	Rhizophyte
*Juncus bulbosus* L.	Elodeid	Rhizophyte
*Sparganium natans* L.	Elodeid	Rhizophyte
*Elatine hydropiper* L.	Isoetid	Rhizophyte
*Elatine orthosperma* Düben	Isoetid	Rhizophyte
*Elatine triandra* Schkuhr	Isoetid	Rhizophyte
*Eleocharis acicularis* (L.) Roem. et Schult.	Isoetid	Rhizophyte
*Isoetes echinospora* Durieu	Isoetid	Rhizophyte
*Isoetes lacustris* L.	Isoetid	Rhizophyte
*Littorella uniflora* (L.) Asch.	Isoetid	Rhizophyte
*Lobelia dortmanna* L.	Isoetid	Rhizophyte
*Ranunculus reptans* L.	Isoetid	Rhizophyte
*Subuluria aquatica* L.	Isoetid	Rhizophyte
*Nuphar lutea* (L.) Sibth. & Sm.	Floating-leaved species	Rhizophyte
*Nuphar lutea x pumila*	Floating-leaved species	Rhizophyte
*Nuphar pumila* (Timm) DC.	Floating-leaved species	Rhizophyte
*Nymphaea alba ssp. alba*	Floating-leaved species	Rhizophyte
*Nymphaea alba ssp. candida* (C. Presl & J. Persl) Korsh	Floating-leaved species	Rhizophyte
*Nymphaea alba ssp. candida x tetragona*	Floating-leaved species	Rhizophyte
*Nymphaea tetragona* Georgi	Floating-leaved species	Rhizophyte
*Persicaria amphibia* (L.) Delarbre	Floating-leaved species	Rhizophyte
*Potamogeton natans* L.	Floating-leaved species	Rhizophyte
*Sagittaria natans* Pall.	Floating-leaved species	Rhizophyte
*Sparganium angustifolium* Michx.	Floating-leaved species	Rhizophyte
*Sparganium gramineum* Georgi	Floating-leaved species	Rhizophyte
*Sagittaria natans x sagittifolia*	Floating-leaved species	Rhizophyte
*Alisma plantago-aquatica* L.	Helophyte	Helophyte
*Alopecurus aequalis* Sobol.	Helophyte	Helophyte
*Butomus umbellatus* L.	Helophyte	Helophyte
*Eleocharis mamillata* (H. Lindb.) H. Lindb. ex Dörfl.	Helophyte	Helophyte
*Eleocharis palustris* (L.) Roem. et Schult.	Helophyte	Helophyte
*Equisetum fluviatile* L.	Helophyte	Helophyte
*Glyceria fluitans* (L.) R. Br.	Helophyte	Helophyte
*Hippuris vulgaris* L.	Helophyte	Helophyte
*Iris pseudacorus* L.	Helophyte	Helophyte
*Lysimachia thyrsiflora* L.	Helophyte	Helophyte
*Phragmites australis* (Cav.) Trin. ex Steud.	Helophyte	Helophyte
*Ranunculus lingua* L.	Helophyte	Helophyte
*Sagittaria sagittifolia* L.	Helophyte	Helophyte
*Schoenoplectus lacustris* (L.) Palla	Helophyte	Helophyte
*Scolochloa festucacea* (Willd.) Link.	Helophyte	Helophyte
*Sparganium emersum* Rehmann	Helophyte	Helophyte
*Sparganium erectum* L.	Helophyte	Helophyte
*Typha angustifolia* L.	Helophyte	Helophyte
*Typha latifolia* L.	Helophyte	Helophyte
*Bidens cernua* L.	Shore plant	Shore plant
*Bidens radiata* Thuill.	Shore plant	Shore plant
*Bidens tripartita* L.	Shore plant	Shore plant
*Calla palustris* L.	Shore plant	Shore plant
*Caltha palustris* L.	Shore plant	Shore plant
*Carex acuta* L.	Shore plant	Shore plant
*Carex aquatilis* Wahlenb.	Shore plant	Shore plant
*Carex diandra* Schrank	Shore plant	Shore plant
*Carex elata* All.	Shore plant	Shore plant
*Carex lasiocarpa* Ehrh.	Shore plant	Shore plant
*Carex paniculata* L.	Shore plant	Shore plant
*Carex pseudocyperus* L.	Shore plant	Shore plant
*Carex rostrata* Stokes	Shore plant	Shore plant
*Carex vesicaria* L.	Shore plant	Shore plant
*Cicuta virosa* L.	Shore plant	Shore plant
*Cladium mariscus* (L.) Pohl	Shore plant	Shore plant
*Comarum palustre* L., *Potentilla palustris* (L.) Scop.	Shore plant	Shore plant
*Juncus filiformis* L.	Shore plant	Shore plant
*Lycopus europaeus* L.	Shore plant	Shore plant
*Lysimachia vulgaris* L.	Shore plant	Shore plant
*Lythrum salicaria* L.	Shore plant	Shore plant
*Menyanthes trifoliata* L.	Shore plant	Shore plant
*Phalaris arundinacea* L.	Shore plant	Shore plant
*Rumex aquaticus* L.	Shore plant	Shore plant
*Scirpus sylvaticus* L.	Shore plant	Shore plant
*Thelypteris palustris* Schott	Shore plant	Shore plant
*Chara globularis* Thuillier	Charophyte	Rhizophyte
*Nitella confervacea* A. Braun	Charophyte	Rhizophyte
*Nitella flexilis* (L.) Agardh	Charophyte	Rhizophyte
*Nitella opaca* (Bruzelius) Agardh	Charophyte	Rhizophyte
*Nitella wahlbergiana* Wallman	Charophyte	Rhizophyte

We used different lake characteristics related to water quality and hydro-morphology as explanatory variables (Table 2). Because sampling effort varied with lake size, we used the total transect length of the lake to control for the effects of sampling. We estimated habitat area for aquatic macrophytes as Secchi depth, reflecting water color and turbidity, and thus the vertical amount of space available. Also, we included shoreline development factor SDF = shoreline length/[2 × (π × surface area of the lake)∧(½)] as a measure of habitat area indicating the complexity of the shoreline (i.e., habitat heterogeneity) (Dodds, 2002). Water quality variables included total phosphorus (TP), which is usually considered as a limiting nutrient for primary productivity in boreal freshwaters (Wetzel, 2001), pH, alkalinity and conductivity. As pH, alkalinity and conductivity were highly intercorrelated (conductivity and pH: Rp = 0.740, *P* < 0.001; conductivity and alkalinity: Rp = 0.936, *P* < 0.001; pH and alkalinity Rp = 0.806, *P* < 0.001) we used principal component analysis to extract one principal component (i.e., pH, alkalinity and conductivity, “PAC”). This component explained 88.58% of the total variance.

**Table 2:** Environmental characteristics of the study lakes.

**Variable**	**Status**	**Mean**	**95% CI's**		**Differing groups**
Total line length (m)	High	869	663	1,075	
	Good	748	536	959	
	Moderate	882	691	1,074	
Shoreline Development Factor (SDF)	High	2.8	2.3	3.3	
	Good	3.6	2.9	4.2	
	Moderate	3.0	2.4	3.6	
Secchi depth (m)	High	2.91	2.26	3.55	H & G > M
	Good	2.02	1.72	2.32	
	Moderate	1.32	1.12	1.51	
Total phosphorus (mg P m^−3^)	High	11.91	8.276	15.543	H & G < M
	Good	18.93	15.539	22.318	
	Moderate	41.78	30.835	52.730	
pH	High	6.9	6.6	7	
	Good	6.8	6.5	6.9	
	Moderate	7.5	6.3	7.8	
Alkalinity (mmol l^−1^)	High	0.110	0.082	0.138	H & G < M
	Good	0.119	0.089	0.149	
	Moderate	0.334	0.258	0.409	
Conductivity (mS cm^−1^)	High	3.139	2.426	3.852	H & G < M
	Good	3.498	2.868	4.128	
	Moderate	7.165	5.619	8.712	

*Means and 95% Confidence Intervals (CIs) within lakes classified as in high (H), good (G) or moderate (M) status. In a case that groups' 95% CIs do not cross, it is indicated which of the groups differ*.

We obtained shoreline length and water area from a lake morphology database of the Finnish Environment institute (https://www.avoindata.fi), and water quality data from the Finnish national database of surface water quality (http://www.syke.fi/en-US/Open_information). The database contains measurements conducted using standardized sampling procedures and quality controlled laboratory protocols. Values of TP, alkalinity, conductivity and pH are sample medians of the surface layer (0–2 m) of the deepest area of each lake during the growing season (1.6–30.9) of primarily 2000–2007. Secchi depth measurements are from the same period and sampling sites.

To determine the level of human impact we used the integrated assessment of ecological status (*sensu* the EU Water Framework Directive) of surface waters in Finland. The classification, led by the Finnish Environment Institute, thoroughly evaluates the ecological status of a lake and has been conducted twice: in 2008 and in 2013 (Vuori et al., 2009; Rask et al., 2011; Aroviita et al., 2012; Andersen et al., 2016). It is based on (1) basic chemical features of the water, (2) occurrence or abundance of certain groups of aquatic organisms (i.e., phytoplankton, periphytic algae, aquatic macrophytes, benthic macroinvertebrates, and fishes), and (3) the degree of human pressure, which is evaluated with multiple criteria such as anthropogenic actions within a drainage basin (i.e., agriculture, industry, forestry, settlements etc. The aquatic macrophyte data used here have also been used in part in the development of the ecological classification of the lakes (Kanninen et al., 2009). Thus, it is expected *a priori* that the lakes classified into different status classes differ in total phosphorus content and show dissimilar communities. However, our detailed research questions (see above) are not considered in the status classification process.

Originally, the lakes were classified as in “high” (21 lakes), “good” (22 lakes), “moderate” (21 lakes), and “poor” (2 lakes) status. As only two lakes in the data set were classified as “poor” they were pooled into “moderate,” resulting in 23 lakes for this class. We chose to retain the two lakes because they add valuable information, and excluded them only from the analyses where differences in group size affect the results (see *Statistical Analyses*). As expected, mean values of Secchi depth, TP, pH, alkalinity and conductivity differed between the lake groups (Table 2).

### Statistical analyses

We used generalized least square models to analyze the relationship between species richness and different explanatory variables, for each of the five dependent variables separately. We developed a complete set of 31 models including all explanatory variable (total transect length; Secchi depth; shoreline development factor: SDF; total phosphorus: TP; a compound variable for pH, alkalinity and conductivity: PAC; status) combinations. We always included total transect length to control for the effect of sampling. All dependent and continuous explanatory variables, except PAC, were log_10_-transformed [log_10_(*n*+1) for free-floating species richness] prior to analysis to normalize residuals, and also because we expected species richness to have a linear log-log relationship with the explanatory variables. We used AICc (Akaike Information Criteria for small sample sizes) to compare alternative models: the model with the smallest AICc is considered to be best with respect to expected Kullback-Leibler information lost (Burnham and Anderson, 2002). Since spatial autocorrelation may violate the assumption of residual independence (Legendre and Legendre, 1998) we investigated whether dependent variables showed significant spatial correlation structures following Zuur et al. (2009, p. 90–92). We observed a significant spatial correlation structure only for total species richness and rhizophytes. Hence, we used a suitable spatial correlation structure when modeling total species richness and rhizophytes (namely the rational and the gaussian correlation structure, respectively). We also analyzed whether species richness (total and the four species groups) differed in lakes with high, good and moderate status by one-way ANOVA. Species richness was interpreted to differ if the 95% Confidence Intervals did not cross.

Differences in community composition and beta diversity can arise through nestedness (resulting from species richness differences) and turnover (where one species replaces another with no change in richness) (Baselga, 2010). We can easily partition between the two by using null models. This allows us to dissect the effect of species richness on community composition and beta diversity, and draw inferences on the processes behind the observed patterns, i.e., whether the observed changes in community composition and beta diversity are solely due to changes in species richness representing neutral processes or independent of species richness, representing selection. We tested the null hypothesis that the observed community composition can be explained by random distribution of species. Our null model is a standard fixed-fixed null model where both the observed species richness as well as the species occurrence were held constant while letting species identities shuffle within these constraints (Gotelli and Graves, 1996). It assumes no dispersal limitation which is a quite plausible assumption since aquatic macrophytes have very high dispersal capacity, at least at the regional scale used here (Viana et al., 2016). First, we defined the regional species pool as the total number of species and their total occurrence rate observed. Then, we calculated observed dissimilarity between each pair of sites using incidence-based Sørensen's dissimilarity index. Next, we calculated expected Sørensen's dissimilarity between each pair of sites by randomly sampling species from the regional species pool while preserving the relative occupancy of each species and observed species richness in each site. From 999 iterations we calculated the standardized effect size (dissimilarity deviation) as the difference between observed dissimilarity and mean expected dissimilarity divided by the standard deviation of the expected values. If dissimilarity deviation is lower than zero it indicates that observed dissimilarity is lower than expected by random chance, whereas deviation larger than zero indicates dissimilarity higher than expected by random chance. If any of the study variables had an effect on dissimilarity deviation, this would indicate that processes other than neutral random sampling e.g., deterministic processes are responsible for changes in community composition.

**Figure 2:**
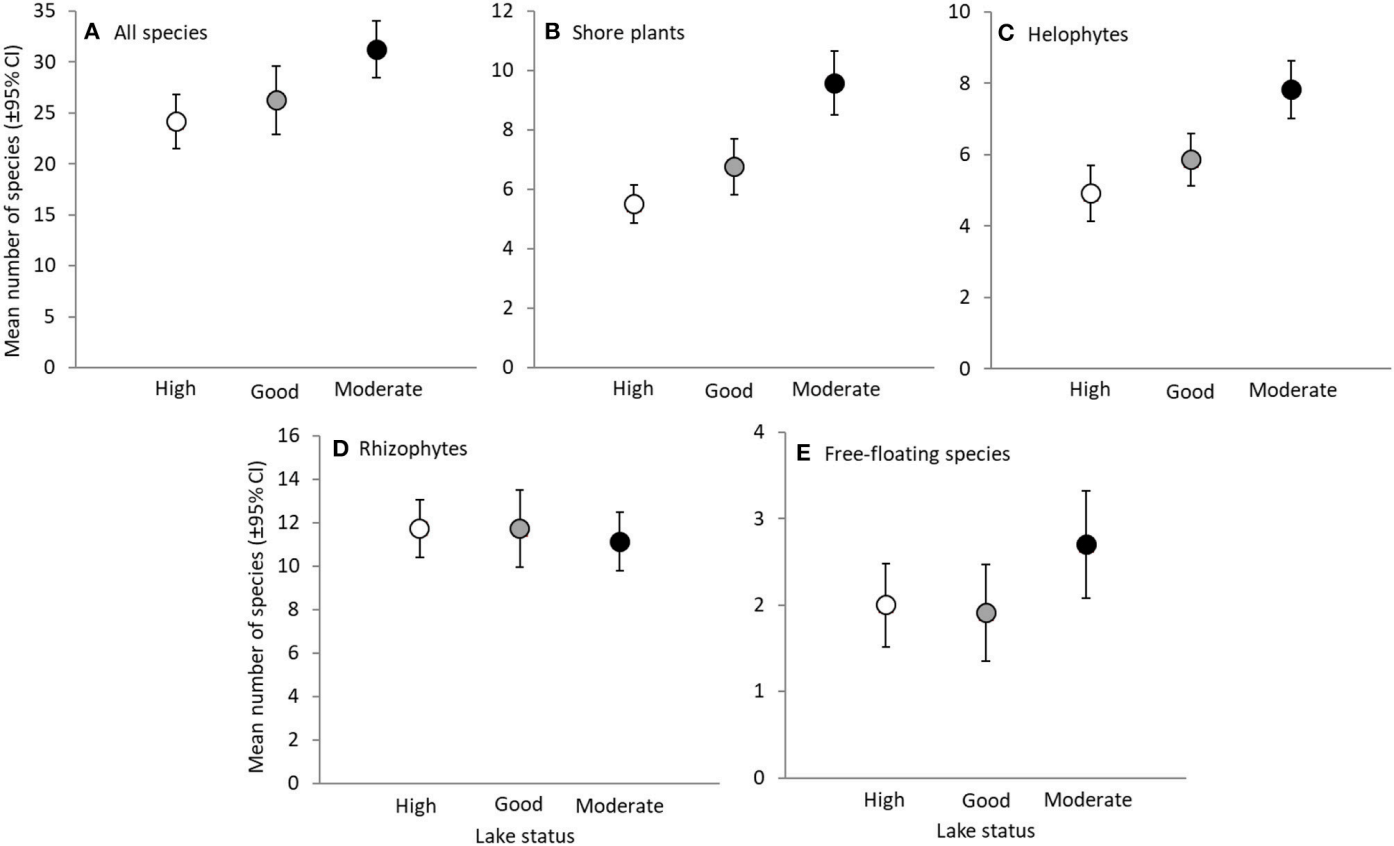
Species richness of different species groups in lakes with high, good and moderate status. Mean number of species (±95% Confidence Intervals) of all species **(A)**, shore plants **(B)**, helophytes **(C)**, rhizophytes **(D)** and free-floating species **(E)**. Note the different scaling of y-axes.

We tested whether different lake groups (high, good, moderate human impact) have different (i) mean community composition, and/or (ii) beta diversity (i.e., dispersion in community composition). They can be visually interpreted for instance in Figure 2A where mean community composition is the location of the center of a given group whereas beta diversity is the spread of the individual data points around the center. We expected *a priori* that the mean community composition of different lake groups is different, and used permutational multivariate analysis of variance (PERMANOVA) to confirm this. To test whether beta diversity differed among the three groups we used the distance-based test for homogeneity of multivariate dispersions (Anderson et al., 2006). It is essentially a multivariate extension of Levene's test of homogeneity and counts the distance of each site to multivariate centroid of the group (Anderson et al., 2006). Both PERMANOVA and the distance-based test for homogeneity of multivariate dispersions are nonparametric and statistical significance was tested by 999 permutations (Anderson, 2001; Anderson et al., 2006). Both analyses were run separately on observed dissimilarity and dissimilarity deviation. These methods are sensitive to variation in group size, and thus the two lakes that we originally classified as into poor status were omitted. As habitat heterogeneity is often shown to increase beta diversity (Alahuhta et al., 2017), we also tested whether the environmental variables showed homogeneity of variance within all of the three groups.

We used multivariate distance matrix regression (MDMR; Anderson, 2001; McArdle and Anderson, 2001; McArtor et al., 2017) to test for the effect of each environmental variable on both observed dissimilarity and dissimilarity deviation. MDMR is very similar to the widely used ordination method Redundancy Analysis. On the contrary to Redundancy Analysis which uses solely Euclidian distances, MDMR can be based on any dissimilarity measure. Moreover, rather than relying on permutation-based *p*-values MDMR provides analytical *p*-values (McArtor et al., 2017). These are provided for all predictors jointly (omnibus effect) and for each predictor individually, conditioned on the rest (McArtor et al., 2017), which makes comparison of the individual variables easier. To control for possible bias arising from the possibility that some spatially autocorrelated unmeasured environmental variables or dispersal may affect community composition we used distance-based redundancy analysis (Legendre and Anderson, 1999) to search the spatial variables which best explained the variation in the data by forward selection of the available spatial variables. These spatial variables were geographic coordinates of the sites (latitude, longitude) and the spatial eigenfunctions with positive eigenvalues (i.e., positively autocorrelated) from the principal components of neighbor matrices (Borcard and Legendre, 2002). The number of selected spatial variables ranged from 3 to 6 and they were used as additional explanatory variables.

All the analyses were performed with R version 3.0.3 (R Foundation for Statistical Computing, Vienna, AT) with packages CompQuadForm (Duchesne and Lafaye De Micheaux, 2010), MDMR (McArtor, 2016), MuMIn (Barton, 2015), nmle (Pinheiro et al., 2013), and vegan (Oksanen et al., 2013).

## Results

### Species richness

The chemical properties of the water were important drivers of total species richness (Table 3). The compound factor for pH, alkalinity and conductivity (PAC) and total transect length were strongly and positively associated with all measures of species richness (Table 3). These two most important variables (PAC and total transect length) were accompanied by others depending on the species group studied: total species richness as well as species richness of shore plants, helophytes and rhizophytes were clearly higher in lakes with high shoreline development factor (SDF). That is, the lakes with complex shoreline had higher species richness. Total phosphorus (TP) was positively related especially to shore plant species richness. Secchi depth was negatively related to helophyte species richness but overall its effect was relatively weak and the sign depended on the studied species group.

**Table 3:** Generalized least square models between species richness (all species, shore plants, helophytes, rhizophytes and free-floating species separately) and different explanatory variables [transect length, Secchi depth, shoreline development factor (SDF), total phosphorus (TP), a combined variable for pH, alkalinity and conductivity (PAC), lake status (high, good, moderate)].

	**df**	***L***	**AICc**	***d***	***w***	**Intercept**	**Transect lenght**	**Secchi debth**	**SDF**	**TP**	**PAC**	**Status**
All species	8	71.1	−151.1	0.0	0.46	0.35	0.32		0.19	0.05	0.06	
Shore plants	6	47.4	−107.4	0.0	0.58	−0.15	0.20		0.30	0.22	0.07	
	7	47.0	−106.1	1.3	0.30	−0.32	0.20	0.16	0.31	0.33	0.06	
Helophytes	6	43.5	−99.0	0.0	0.17	−0.23	0.24		0.15	0.20	0.06	
	7	41.7	−98.3	0.6	0.13	0.00	0.27	−0.20			0.05	x
	6	43.4	−98.0	1.0	0.10	0.09	0.24	−0.28	0.14		0.07	
	5	43.4	−97.8	1.2	0.10	−0.20	0.25			0.21	0.05	
	7	41.1	−97.5	1.5	0.08	−0.22	0.26			0.14	0.05	x
	5	43.5	−97.2	1.7	0.07	0.13	0.25	−0.29			0.06	
	8	40.8	−97.2	1.8	0.07	−0.01	0.26	−0.21	0.09		0.06	x
Rhizophytes	7	50.0	−103.8	0.0	0.11	−0.26	0.43		0.12		0.03	
	8	49.3	−103.4	0.4	0.09	−0.30	0.43	0.12	0.15		0.03	
	5	52.4	−103.3	0.5	0.09	−0.20	0.42					
	6	50.2	−103.1	0.8	0.08	−0.21	0.43				0.02	
	6	51.4	−102.6	1.3	0.06	−0.23	0.42		0.09			
	8	47.1	−102.1	1.7	0.05	−0.19	0.42				0.03	x
Free-floating species	4	24.0	−39.3	0.0	0.29	−0.50	0.34				0.08	
	5	24.5	−38.0	1.4	0.15	−0.44	0.35			−0.07	0.09	
	6	25.5	−37.6	1.8	0.12	−0.05	0.35	−0.36		−0.30	0.10	

*Degrees of freedom (df), likelihood (L), Akaike Information Criteria (AICc), distance from the best model (d), and Akaike weight (w) are shown for all the models with d < 2*.

As expected, total species richness differed between lakes depending on their status [one-way ANOVA: *F*_(df1, df2)_ = 6.01_(2, 63)_, *P* = 0.004]. Lakes with moderate status had higher total species richness than lakes with high status, and higher richness of shore plants and helophytes than lakes with high or good status (Figure 2).

### Community composition

**Figure 3:**
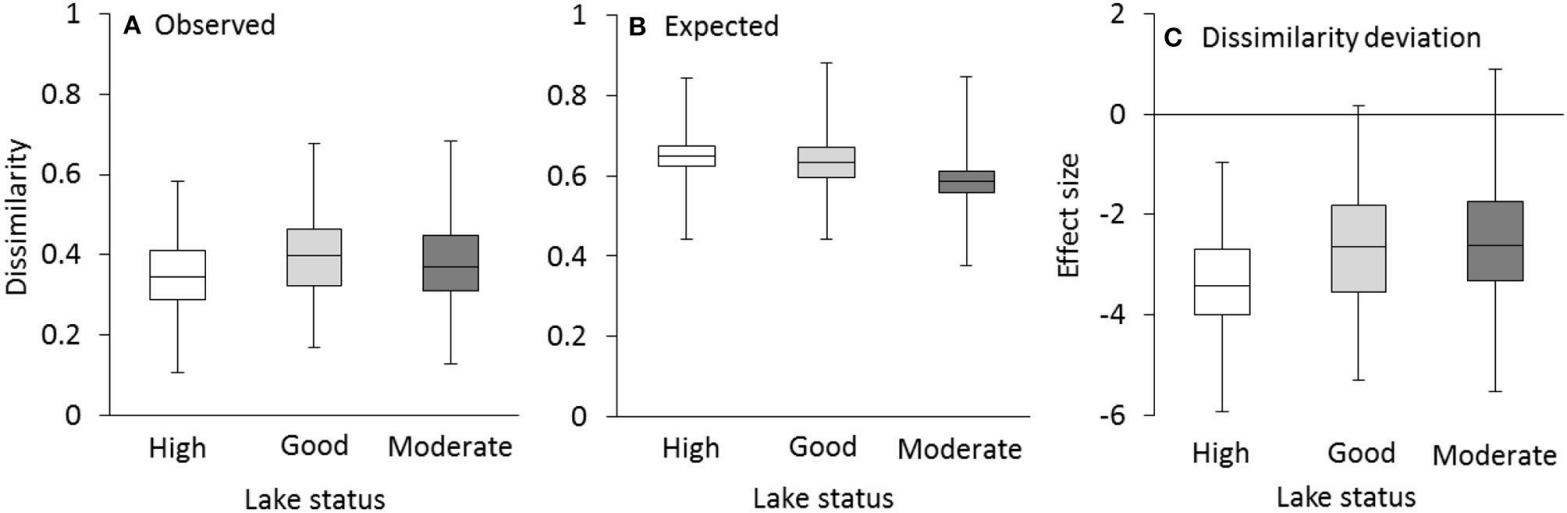
Observed within-group dissimilarities in community composition were smaller than expected on the basis of the null model resulting in negative dissimilarity deviations. Observed Sørensen's dissimilarity **(A)**, expected dissimilarity **(B)**, and effect size **(C)** for all species in lakes with high, good and moderate status. Box-plots represent minimum, second quantile, median, third quantile and maximum values.

Observed dissimilarities in community compositions within groups were consistently lower than null model expectations, generally leading to negative dissimilarity deviations (Figure 3). That is, communities within lakes having the same status were more similar than what could be expected by random sampling. The difference between observed and expected community composition was more pronounced in lakes with high status where even the maximum values of dissimilarity deviations were below zero.

The environmental variables studied affected clearly the community composition. The combined effect of all variables (omnibus effect) for all species groups studied was highly significant (Table 4). Shoreline development factor (SDF) affected observed dissimilarity among lakes of community composition of all species, shore plants and helophytes but not that of rhizophytes or free-floating species (Table 4). Observed dissimilarities of all studied species groups were affected by PAC whereas SDF was important for all species, shore plants and helophytes (Table 4). Secchi depth affected observed dissimilarities of helophytes and TP observed dissimilarities of all species and helophytes (Table 4). Mean community composition differed among lakes with different status (Table 5). This was true for each of the studied species groups and for both observed dissimilarity and dissimilarity deviation. Thus, besides having higher species richness, the lakes with moderate status had also different community composition (reflecting not only species richness).

**Table 4:** Associations between aquatic macrophyte community composition and environmental variables.

**Dependent variable**	**Effect**	**Observed dissimilarity**				**Dissimilarity deviation**			
		**Statistic**	**df**	**Pseudo *R*^2^**	***P***	**Statistic**	**df**	**Pseudo *R*^2^**	***P***
All species	Omnibus	**0.95**	**11**	**0.49**	**<0.002**	**1.11**	**11**	**0.53**	**<0.002**
	Total line length	**0.07**	**1**	**0.04**	**0.004**	**0.06**	**1**	**0.03**	**0.01**
	sDepth	0.02	1	0.01	0.212	0.02	1	0.01	0.278
	SDF	**0.05**	**1**	**0.03**	**0.018**	**0.04**	**1**	**0.02**	**0.048**
	TP	0.03	1	0.01	0.178	**0.05**	**1**	**0.02**	**0.028**
	PAC	**0.09**	**1**	**0.05**	**<0.002**	**0.1**	**1**	**0.05**	**<0.002**
	Status	0.02	1	0.01	0.35	0.02	1	0.01	0.29
	PCNM3	0.01	1	0.01	0.782	0.02	1	0.01	0.54
	PCNM6	**0.04**	**1**	**0.02**	**0.036**	**0.05**	**1**	**0.02**	**0.036**
	PCNM24	**0.04**	**1**	**0.02**	**0.046**	**0.05**	**1**	**0.02**	**0.016**
	N	0.03	1	0.02	0.114	**0.07**	**1**	**0.03**	**<0.002**
	PCNM2^*^/E^**^	0.03	1	0.02	0.11	0.04	1	0.02	0.068
Shore plants	Omnibus	**0.82**	**9**	**0.45**	**<0.002**	**0.69**	**9**	**0.41**	**0.002**
	Total line length	**0.08**	**1**	**0.05**	**0.002**	**0.05**	**1**	**0.03**	**0.036**
	sDepth	0	1	0	0.844	0.01	1	0.01	0.718
	SDF	**0.14**	**1**	**0.08**	**<0.002**	**0.1**	**1**	**0.06**	**0.002**
	TP	0.02	1	0.01	0.244	0.02	1	0.01	0.316
	PAC	**0.06**	**1**	**0.03**	**0.032**	**0.07**	**1**	**0.04**	**0.014**
	Status	0.02	1	0.01	0.412	0.01	1	0.01	0.592
	PCNM3	0.01	1	0	0.69	0.02	1	0.01	0.448
	PCNM6	0.04	1	0.02	0.074	**0.05**	**1**	**0.03**	**0.038**
	PCNM2^*^/N^**^	0.05	1	0.03	0.058	**0.06**	**1**	**0.03**	**0.014**
Helophytes	Omnibus	**0.85**	**9**	**0.46**	**<0.002**	**0.69**	**10**	**0.41**	**0.002**
	Total line length	**0.13**	**1**	**0.07**	**<0.002**	**0.09**	**1**	**0.05**	**0.002**
	sDepth	**0.06**	**1**	**0.03**	**0.02**	**0.05**	**1**	**0.03**	**0.042**
	SDF	**0.06**	**1**	**0.04**	**0.018**	0.04	1	0.02	0.096
	TP	**0.07**	**1**	**0.04**	**0.014**	0.05	1	0.03	0.068
	PAC	**0.08**	**1**	**0.04**	**0.006**	**0.06**	**1**	**0.03**	**0.012**
	Status	0.01	1	0.01	0.51	0.01	1	0.01	0.506
	PCNM3	0.01	1	0.01	0.508	0.02	1	0.01	0.262
	PCNM6	0.04	1	0.02	0.11	0.05	1	0.03	0.042
	PCNM2^*^/PCNM7^**^	0.03	1	0.02	0.202	0.05	1	0.03	0.05
	N^**^					**0.05**	**1**	**0.03**	**0.038**
Rhizophytes	Omnibus	**0.92**	**11**	**0.48**	**<0.002**	**0.99**	**12**	**0.5**	**<0.002**
	Total line length	0.04	1	0.02	0.056	0.03	1	0.02	0.142
	sDepth	0.01	1	0	0.82	0.01	1	0	0.746
	SDF	0.01	1	0.01	0.758	0	1	< 2e−16	0.994
	TP	0.04	1	0.02	0.104	0.03	1	0.01	0.158
	PAC	**0.09**	**1**	**0.05**	**<0.002**	**0.08**	**1**	**0.04**	**<0.002**
	Status	0.03	1	0.01	0.26	0.03	1	0.01	0.184
	PCNM1	**0.05**	**1**	**0.03**	**0.016**	**0.06**	**1**	**0.03**	**0.022**
	PCNM6	**0.05**	**1**	**0.03**	**0.018**	**0.06**	**1**	**0.03**	**0.018**
	PCNM13	0.04	1	0.02	0.058	0.04	1	0.02	0.074
	PCNM24	**0.05**	**1**	**0.02**	**0.026**	**0.06**	**1**	**0.03**	**0.02**
	E	**0.07**	**1**	**0.04**	**0.004**	**0.06**	**1**	**0.03**	**0.008**
	PCNM5^**^					0.01	1	0.01	0.678
Free-floating species	Omnibus	**0.71**	**10**	**0.42**	**<0.002**	**1.03**	**12**	**0.51**	**<0.002**
	Total line length	0	1	0	0.91	0.02	1	0.01	0.406
	sDepth	0.03	1	0.02	0.302	0.02	1	0.01	0.388
	SDF	0.02	1	0.01	0.492	0.01	1	0	0.664
	TP	0.01	1	0	0.806	0.01	1	0.01	0.584
	PAC	**0.08**	**1**	**0.05**	**0.014**	**0.09**	**1**	**0.05**	**0.006**
	Status	0.02	1	0.01	0.32	0.05	1	0.03	0.102
	PCNM6	0.02	1	0.01	0.416	0.02	1	0.01	0.52
	PCNM8	**0.09**	**1**	**0.05**	**0.01**	**0.08**	**1**	**0.04**	**0.006**
	E	**0.06**	**1**	**0.04**	**0.048**	**0.08**	**1**	**0.04**	**0.036**
	N	0	1	0	0.806	0.02	1	0.01	0.494
	PCNM21^**^					0.04	1	0.02	0.15
	PCNM27^**^					**0.12**	**1**	**0.06**	**0.002**

Multivariate distance matrix regression MDMR results for dissimilarity test statistic, pseudo R^2^ statistic, and analytic p-values for all predictors jointly (omnibus effect) and for each predictor individually, conditioned on the rest. The background of the effect is shaded if P < 0.05. PCNM variables are the spatial eigenfunctions with positive eigenvalues from the Principal Components of Neighbor Matrices; N, latitude; E, longitude.

* only for observed dissimilarity

** *only for dissimilarity deviation. Statistically significant effects (p < 0.05) are indicated in bold*.

**Table 5:** Lakes in different status groups (high, good or moderate) show different mean community composition (PERMANOVA) but not different dispersion in community composition (Homogeneity of multivariate dispersion).

**Dependent variable**		**PERMANOVA**		**Dispersion**	
		***F*_df1, df2_**	***P***	***F*_df1, df2_**	***P***
Observed dissimilarity	All species	**16.5**_1,62_	<**0.001**	1.2_2,61_	0.308
	Shore plants	**17.8**_1,62_	<**0.001**	1.0_2,61_	0.388
	Helophytes	**13.6**_1,62_	<**0.001**	0.8_2,61_	0.454
	Rhizophytes	**15.8**_1,62_	<**0.001**	2.3_2,61_	0.104
	Free-floating species	**9.7**_1,56_	<**0.001**	0.1_2,55_	0.966
Dissimilarity deviation	All species	**19.6**_1,62_	<**0.001**	2.4_2,61_	0.083
	Shore plants	**15.1**_1,62_	<**0.001**	2.9_2,61_	0.066
	Helophytes	**9.3**_1,62_	<**0.001**	0.8_2,61_	0.435
	Rhizophytes	**18.3**_1,62_	<**0.001**	2.5_2,61_	0.089
	Free-floating species	**10.9**_1,56_	<**0.001**	0.1_2,55_	0.949

*Statistically significant effects (p < 0.05) are indicated in bold*.

The results for observed dissimilarity and dissimilarity deviation were highly similar (Table 4). This means that the changes in community composition were not only due to changes in species richness. Leaving aside slight differentiation in included spatial variables, there were only two clear differences. For all species there was a significant effect of total phosphorus (TP) on dissimilarity deviation but not on observed dissimilarity. For helophytes, effects of shoreline development factor (SDF) and TP were significant on observed dissimilarity but not on dissimilarity deviation (Table 4). Thus, the effects of SDF and TP on community composition were solely due to the sampling effect. Comparing the associations of species richness and environmental variables to those between community composition and environmental variables showed that the factors that strongly affected species richness affected also community composition, independently of species richness (Tables 3, 4).

### Beta diversity

**Figure 4:**
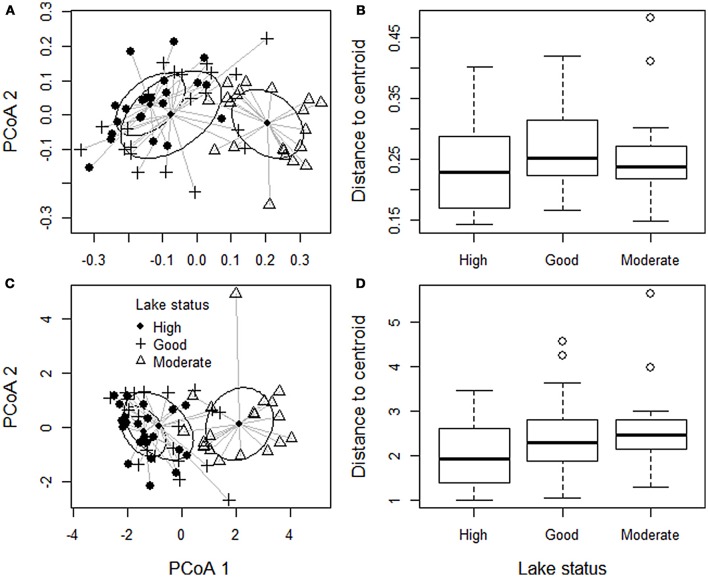
Community composition and beta diversity (i.e., dispersion in community composition within a group) of all macrophyte species. Each point refers to one site. **(A)** Shows the dissimilarity of community composition measured as Sørensen dissimilarity. Although lakes belonging different status groups have different mean community composition (the location of the 1 SD ellipse) beta diversity of each group (i.e., distances to the group centroid) do not differ **(B)**. **(C)** Shows the dissimilarity of community composition measured as dissimilarity deviance, and **(D)** shows distances to centroid for dissimilarity deviance.

The lake groups did not differ in their community dispersion, i.e., beta-diversity, on the basis of distance-based tests for homogeneity of multivariate dispersion (Figure 4, Table 5). Hence, lakes with relatively high human impact were as similar to each other as the lakes with lower human impact. By contrast, lake groups differed in the variation with regards to Secchi depth, TP and PAC [Levene statistic = 6.965_(2, 61)_, *P* = 0.002; Levene statistic = 8.343_(2, 61)_, *P* = 0.001; Levene statistic = 3.957_(2, 61)_, *P* = 0.024; respectively], but not for total transect length (Levene statistic = 0.019_(2, 61)_, *P* = 0.981] or SDF [Levene statistic = 0.068_(2, 61)_, *P* = 0.934]. Thus, lakes with moderate status had higher variance of TP and PAC and lower variance of Secchi depth, in addition to higher mean values of TP and PAC and lower mean values of Secchi depth (Table 2).

## Discussion

Aquatic macrophyte species richness and community composition were affected basically by the same environmental factors, and their relative importance varied according to life-form group under observation. The differences in community composition deviated significantly from the expected random distribution of species indicating deterministic processes. Species richness was higher in lakes with moderate status compared to the lakes with high or good status, and also community composition differed among status, as expected. By contrast, beta diversity did not differ among the lakes under different level of human impact. This suggests that human impact did not lead in homogenization of aquatic macrophyte communities in the study lakes, which are naturally relatively oligotrophic compared to the lakes in many other parts of the world (Elser et al., 2007). We discuss the possible reasons for this finding after concentrating on the results concerning species richness and community composition.

### Species richness

Generally, the most important variables for total species richness were shoreline development factor (SDF) and a combined variable including pH, alkalinity and conductivity (PAC). Also Secchi depth and total phosphorus (TP) were important for specific species groups. The positive effect of lake area to aquatic macrophyte species richness has been recorded in several studies (Rorslett, 1991; Dodson et al., 2000; Jones et al., 2003) and is often explained through habitat heterogeneity: as larger lakes encompass more microhabitats, more species are able to find a suitable habitat with increasing area (Rorslett, 1991; Toivonen and Huttunen, 1995). An especially important factor to the richness of shore plants was SDF: they are restricted solely to shores. In addition, SDF was related also to helophytes and rhizophytes growing further away from the shoreline as these also benefit from the shelter and different microhabitats provided by a more heterogeneous shoreline.

A combined variable including pH, alkalinity and conductivity (PAC) appeared as a highly important factor for species richness for all life-form groups, as was expected on the basis of previous studies (e.g., Vestergaard and Sand-Jensen, 2000; Alahuhta, 2015). These characteristics of water quality are likely to be linked to the amount and form of carbon usable for macrophytes (Toivonen and Huttunen, 1995; Vestergaard and Sand-Jensen, 2000). In boreal lake systems, conductivity reflects the overall trophic state of a lake (Toivonen and Huttunen, 1995). Similarly, the total amount of phosphorus increased particularly shore plant species richness. It is typically the limiting nutrient in freshwaters (Wetzel, 2001). In Finnish lakes increased amount of total phosporus typically increases species richness (Leka et al., 2008) but at very high concentrations species richness starts to decrease (Phillips et al., 2016). Rhizophyte species richness increased with increasing Secchi depth, in line with previous studies (Vestergaard and Sand-Jensen, 2000; Alahuhta, 2015). Increased water transparency typically reflects the vertical expansion of habitats suitable for submerged species. By contrast, helophyte species richness decreased with increasing Secchi depth. It may be that hydrophyte species have competition advantage over helophytes in deeper waters where many hydrophytes have their optimal niche.

### Community composition

Observed within-group dissimilarities in community composition were smaller than expected on the basis of the null model. This resulted in negative dissimilarity deviations indicating that differences in community composition were not due to neutral but deterministic processes. Overall, community composition was associated with the same environmental factors as species richness, as observed also by Alahuhta et al. (2013). This result could have been due to the effect of species richness: increased dissimilarity in community composition could arise simply because more species are observed in a lake. Our null model takes into account this effect: in each lake it sampled the number of actually observed species while species identities were allowed to shuffle. Dissimilarity deviation tells whether the observed dissimilarity of community composition is more or less than could be produced by randomly sampling the observed number of species. Because environmental factors affected not only observed dissimilarity but also dissimilarity deviations, the mutual pattern (community composition was associated with the same environmental factors as species richness) was not governed by the effect of species richness. Thus, high values of pH, alkalinity and conductivity allow a higher number of species per lake but also different species from those that occurred in lakes with lower levels of pH, alkalinity and conductivity. The reason is probably associated with different forms of carbon utilized by macrophytes in photosynthesis selecting for different species sets, in addition to increasing the number of species (Toivonen and Huttunen, 1995; Vestergaard and Sand-Jensen, 2000).

Shoreline development factor (SDF) was an important factor for community composition when considering all species. This was due to its importance for shore plant communities: more complex shorelines supported distinct species than less complex ones. Observed community composition of helophytes was also affected but the effect was solely due to SDF's increasing effect on species richness. By contrast, the effect of Secchi depth on helophytes did not depend on species richness.

### Differences of the lakes under different human impact

As expected, species richness was generally higher in lakes with moderate status than in lakes with high or good status, and as expected, lakes in different human-impacted groups showed clearly different community composition. Contrary to our expectations, beta diversity among the groups (high, good, moderate status) did not differ, i.e., communities in lakes with moderate status were no more homogeneous than lakes with high or good status. This may be due to inherent trophic gradient; our lakes were naturally oligotrophic, relatively species poor. Thus, even the lakes with good or high ecological status are meso-eutrophic at most. By contrast, naturally meso-eutrophic lakes might homogenize when they reach hypertrophy due to excessive nutrient leakage (Lougheed et al., 2008). Interestingly, the variables having the strongest effects on community composition (independently of species richness), namely PAC and total phosphorus, were more dispersed in lakes with moderate status than in lakes with high or good status but this did not result in increased beta diversity. Thus, beta diversity in lakes with moderate status was smaller than expected on the basis of the environmental characteristics. It must also be kept in mind that in this study both the measure of community composition as well as the null model is based on presence-absence data. Thus changes in species abundances and consequent potential homogenization over the landscape may have remained undetected.

In the test of homogeneity of multivariate dispersion which was used to analyze beta diversity, the definition of groups has naturally a major effect on the result. To ensure that our result of no difference in beta diversity among levels of human impact is not biased (due to the fact that community composition and status group are not totally independent, as explained in the Methods section), we repeated our analyses with forming the three groups based on total phosphorus (TP) which was an independently measured variable. The results were similar: there were no differences among groups (results not shown). This confirms that our results are not because of the use of ecological classification only.

In these relatively oligotrophic boreal lakes, increases in total phosphorus (TP) concentration resulting in increased productivity is mainly due to agriculture and other human actions within basins. This is tricky as human impact (in its various forms) has shown to decrease beta diversity (Lougheed et al., 2008; Donohue et al., 2009), whereas increased productivity is associated with increases in beta diversity (Chase and Leibold, 2002; Murphy and Romanuk, 2012). Thus, our result of no change in beta diversity contradicts both of these expectations. We suggest that our result of equal beta diversity in lakes stems from two opposing forces. Chalcraft et al. (2008) showed that the effects of nutrient enrichment on beta diversity can be dependent on the initial productivity of the site. Accordingly, nutrient enrichment may increase beta diversity at sites with low initial productivity and low beta-diversity but decrease beta diversity at sites with high initial productivity and high beta-diversity resulting in equal beta diversity levels after eutrophication. Moreover, the lakes may differ in their original community composition. Priority effects are important in determining beta diversity (Chase and Leibold, 2002) and the studied lakes most probable had originally slightly different community composition which in turn affects the identity of the species able to colonize these lakes. Thus, differences in the original community composition may override effects of human impact in beta diversity.

To conclude, our results showed that human impact did not result in homogenization but instead in changes in mean community composition (independently of species richness) and increase in species richness within the studied gradient. The lack of apparent homogenization may be due to the exact gradient studied, as well as differences in the initial trophic stage and community composition in the studied lakes. It is likely that multiple mechanisms leading to contrasting patterns are involved. Moreover, it must be noted that beta diversity in lakes with moderate status was smaller than expected on the basis of the main environmental characteristics shaping community composition. This could be seen as a first sign of homogenization.

## Data availability statement

Datasets are available on request: the raw data supporting the conclusions of this manuscript will be made available by the authors, without undue reservation, to any qualified researcher.

## Author contributions

All authors contributed to the idea of the study. AK, KM, and KS were responsible for the data gathering. ME analyzed the data and led the writing together with JA. All the other authors contributed significantly to the final manuscript.

### Conflict of interest statement

The authors declare that the research was conducted in the absence of any commercial or financial relationships that could be construed as a potential conflict of interest.

## Abbreviations

MDMR: multivariate distance matrix regression
PAC: a component from principal component analysis describing the water ionic and electrical characteristics i.e.,
pH: alkalinity and conductivity
PERMANOVA: Permutational multivariate analysis of variance
SDF: Shoreline development factor
TP: Total amount of phosphorus.
